# The Role of Hormonal Factors in Weight Loss and Recidivism after Bariatric Surgery

**DOI:** 10.1155/2013/528450

**Published:** 2013-10-23

**Authors:** S. D. Pedersen

**Affiliations:** C-ENDO Endocrinology Centre, Suite 240, 1016-68th Avenue SW, Calgary, AB, Canada T2V 4J2

## Abstract

Substantial heterogeneity exists in weight loss trajectories amongst patients following bariatric surgery. Hormonal factors are postulated to be amongst the contributors to the variation seen. Several hormones involved in hunger, satiety, and energy balance are affected by bariatric surgery, with the alteration in hormonal milieu varying by procedure. Limited research has been conducted to examine potential hormonal mediators of weight loss failure or recidivism following bariatric surgery. While hormonal factors that influence weight loss success following gastric banding have not been identified, data suggest that hormonal factors may be involved in modulating weight loss success following gastric bypass. There may be hormonal mediators involved in determining the weight trajectory following sleeve gastrectomy, though the extremely limited data currently available prohibits definitive conclusions from being drawn. There is great need for future research studies to explore this knowledge gap, as improving this knowledge base could be of benefit to guide clinicians toward understanding the hormonal contributors to a patient's postoperative weight loss failure or recidivism or perhaps be of value in selecting the most appropriate bariatric procedure based on the preoperative hormone milieu. Integrative interdisciplinary approaches exploring these complex interrelationships could potentially increase the explanatory power of such investigations.

## 1. Introduction

Bariatric surgery is currently the most effective treatment for severe obesity [1]. While mechanical restriction of food intake is a dominating contributor to the weight loss seen after bariatric surgery, at least initially, weight loss that often exceeds that expected by restriction alone is often seen [2]. There is a growing consensus that hormones play an important part in weight loss induced by bariatric surgery [3].

The extent and sustainability of postsurgical weight loss differ markedly between individuals [4]. This heterogeneity in response is determined by a wide range of factors related to the type of surgery, patient demographics, and psychosocial factors, as well as biological factors that regulate energy intake, storage, and expenditure [5, 6]. The aim of the current paper is to review the available literature regarding the role of hormonal responses to bariatric surgery and examine their contribution to post-surgical weight trajectories, particularly in regard to weight loss failure and weight regain.

## 2. Key Hormones Involved in Hunger and Satiety

### 2.1. Glucagon-Like Peptide-1

Glucagon-like peptide-1 (GLP-1) is a member of the enteroglucagon peptide family and is secreted by the L cells of the distal intestine in response to a meal [7]. Postprandial GLP-1 secretion is associated with an increase in insulin release from pancreatic beta cells while suppressing glucagon release from pancreatic alpha cells (the “incretin effect”). GLP-1 also promotes satiety both through a direct inhibitory effect on gastric emptying and a stimulatory effect on the satiety centre in the paraventricular nucleus of the hypothalamus [7]. Obese humans display an attenuated postprandial GLP-1 response compared to lean subjects, and diet induced weight loss is associated with an increase in postprandial GLP-1 release [8].

Bariatric procedures where the delivery of undigested nutrients occurs more rapidly, or farther down, into the small intestine, are associated with a greater L cell response to release GLP-1 in response to a meal [9]; this is likely to be an important mechanism that contributes to weight reduction seen after these surgical procedures.

### 2.2. Peptide Tyrosine Tyrosine 3-36

Peptide tyrosine tyrosine 3-36 (PYY 3-36) is a peptide that is cosecreted with GLP-1 from the L cells in response to a meal [3]. A precursor is cleaved by the enzyme DPP-4 into the main circulating and active form of PYY, termed PYY 3-36 [2]. While PYY 3-36 does not appear to share the incretin functions of GLP1, PYY 3-36 does work in a synergistic fashion with GLP-1 to slow gastric emptying and stimulate satiety centrally [10]. PYY 3-36 has also been shown to stimulate thermogenesis and increase energy expenditure [11]. Individuals with obesity display an attenuated meal-stimulated PYY response and require a greater caloric load to achieve a postprandial PYY concentration similar to that seen in lean controls [12]. 

As for GLP-1, the postprandial secretion of PYY 3-36 appears to be potentiated following bariatric surgeries that speed the delivery of undigested nutrients further down the intestine [9] and is also thought to be a contributor to the weight loss seen following these surgical procedures. 

### 2.3. Ghrelin

Ghrelin is an orexigenic hormone produced primarily by the fundus and body of the stomach [9]. A rising level causes an increase in hunger and the initiation of feeding, suppression of ghrelin occurring minutes after feeding. The effect of ghrelin is mediated partially by a humoral effect and partially via the vagus nerve. 

Ghrelin levels are lower in obese individuals compared to lean individuals [12]. Diet-induced weight loss increases ghrelin levels, which may be a barrier to sustaining long-term weight loss [13]. Thus, surgeries that decrease ghrelin levels could be advantageous in that ghrelin would not drive feeding behavior to counteract the weight loss. 

### 2.4. Oxyntomodulin

Oxyntomodulin (OXM) is a peptide that is cosecreted with GLP-1 and PYY by the L cells of the intestine in response to a meal [3]. It is thought of as a “weak incretin” from a glycemic standpoint in that it stimulates insulin secretion and glucagon suppression in response to a meal, but binds to the GLP-1 receptor with an affinity 50 times lower than that for GLP-1 itself. Oxyntomodulin has a centrally acting satiety effect as well as an effect to slow gastric emptying, thereby reducing food intake in both lean and obese individuals [14]. OXM has also been shown to increase energy expenditure when administered to overweight or obese humans [15]. Thus, bariatric procedures that increase OXM would be desirable.

### 2.5. Glucose-Dependent Insulinotropic Polypeptide

Glucose-dependent insulinotropic polypeptide (GIP) is a hormone secreted by the K cells of the duodenum and jejunum [3]. GIP is secreted primarily in response to a meal and has an incretin effect as well as effects on lipid metabolism favoring fat deposition [16]. 

Basal and stimulated GIP levels are elevated in obesity and type 2 diabetes; type 2 diabetics are suspected to be resistant to the insulinotropic effects of GIP [17]. While the exact nature of the relationship between GIP and human obesity is still poorly understood [18], theoretically, a bariatric procedure that results in lower GIP levels could be favorable in decreasing fat stores and long term weight maintenance, though it is unclear what the isolated effect of lower GIP would be on glucose homeostasis. 

### 2.6. Bile Acids

Bile acids, which are synthesized by the liver and excreted in bile, have been more recently recognized to have a hormonal effect on weight regulation. They appear to be important signaling molecules in the regulation of energy expenditure via the G-protein-coupled membrane receptor TGR5 and the nuclear receptor farnesoid X (FXR) [3]. Bile acids increase energy expenditure in brown adipose tissue via the stimulation of TGR5 with subsequent increased conversion of the thyroid hormone tetraiodothyronine (T4) to the active form triiodothyronine (T3) [19, 20] and are also involved in the stimulation of GLP-1 release [10, 20]. Bile acid-induced activation of FXR after a meal induces synthesis of the intestinal peptide hormone FGF19, triggering a cascade that enhances mitochondrial metabolism and decreases insulin resistance [21]. 

Postprandial bile acids have been shown to be lower in obese subjects compared to lean controls [22]. Serum bile acids have been shown to be increased following some types of bariatric procedures, with the mechanism responsible for this increase being poorly elucidated [21, 23, 24]. It follows from the above discussion that an increase in bile acids could be favorable in energy homeostasis. 

### 2.7. Leptin

Leptin is a protein produced primarily by adipose tissue and appears to be reflective of total body energy stores proportional to body fat mass [9]. Though leptin acts on the hypothalamus to induce satiety, its role in common human obesity is not clear. Patients with obesity have higher leptin levels and are thought to manifest leptin resistance, in that the satiety inducing effect of leptin is reduced. Leptin levels decrease with lifestyle induced weight loss [25], which is thought to play a role in the adaptive decrease in energy expenditure and thyroid hormones seen with weight loss, thereby promoting weight regain [26]. 

### 2.8. Adiponectin

Another hormone produced by adipose tissue, adiponectin has an inverse relationship with fat stores as well as insulin resistance, with higher levels shown to be protective against the development of T2DM, by way of its anti-inflammatory and insulin sensitizing properties [27, 28]. Lifestyle induced weight loss increases adiponectin levels [29]. Thus, it would be deemed advantageous for a bariatric surgical procedure to be associated with increased adiponectin levels, from the perspective of improving metabolic parameters.

## 3. Hormonal Changes following Bariatric Surgery

**Figure 1:**
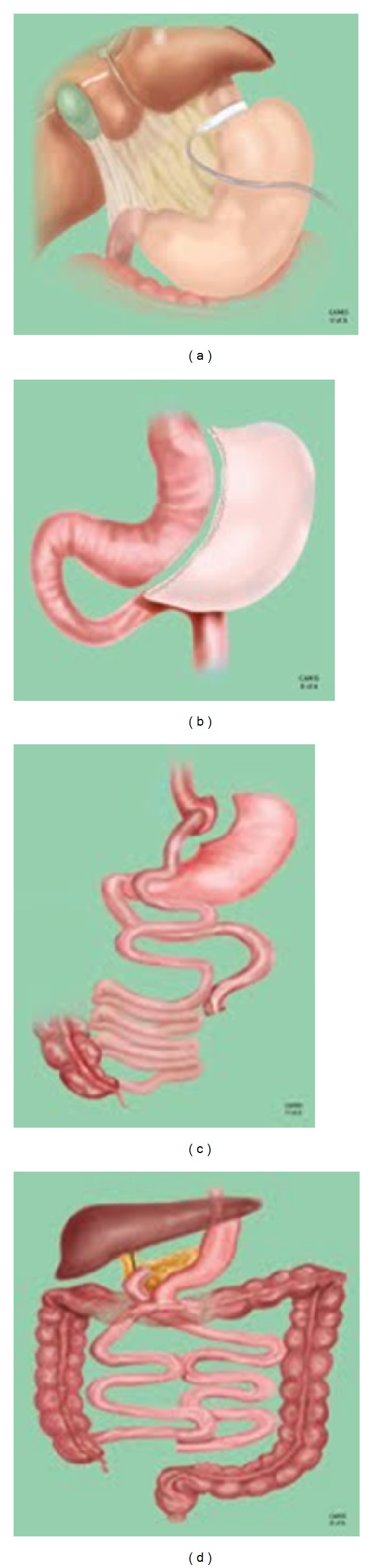
Most commonly performed bariatric surgical procedures. (a) Laparoscopic adjustable gastric banding; (b) sleeve gastrectomy; (c) Roux-en-Y gastric bypass surgery; (d) biliopancreatic diversion with duodenal switch. Reproduced with permission from the Centre for the Advancement of Minimally Invasive Surgery (CAMIS), University of Alberta.

As the various available bariatric surgeries are heterogeneous in terms of the anatomical alterations made, it is important to consider each procedure separately, as the resultant mechanisms involved in weight loss are quite different. The four most commonly undertaken procedures are described below, in order from least to most invasive: laparoscopic adjustable gastric banding, sleeve gastrectomy, Roux-en-Y gastric bypass, and biliopancreatic diversion (Figure 1).

### 3.1. Laparoscopic Adjustable Gastric Banding

Laparoscopic adjustable gastric banding (LAGB) is a procedure in which an inflatable constrictive ring is placed around the gastric inlet (Figure 1(a)). It is considered to be a purely restrictive operation that limits food intake by promoting a vagally mediated sense of satiety [10]. The LAGB provides a profound mechanical effect to restrict food intake, as the band is placed to allow food intake of about a cup of dry food [30]. The LAGB procedure results in approximately 50% of excess body weight loss at 2 years [31].

The effect of LAGB on ghrelin is uncertain [9]. Several studies point to an increase in fasting ghrelin level from 1 day to 2 years postoperatively [32–34], while other studies have found both fasting and meal-suppressed ghrelin levels to be unaffected [35, 36]. Either way, ghrelin does not change in favor of weight loss following LAGB. 

As LABG does not accelerate the delivery of nutrients to the small intestine, it would not be expected that this procedure would have an effect on satiety hormones secreted from the intestine. Accordingly, most studies have demonstrated no effect on GLP-1, PYY 3-36 [35, 36], or GIP [37]. Administration of somatostatin (which nonspecifically suppresses the release of hormones such as GLP-1, PYY, and oxyntomodulin) to patients after LAGB does not affect caloric intake nor feeling of fullness after eating, suggesting that these hormones are not players in LAGB-induced weight loss [38]. Bile acids are either unchanged or lower following LAGB based on very limited available data [20].

As with weight loss of any cause, leptin levels are seen to be decreased as soon as 2 weeks after LAGB; studies done up to 2 years postoperatively have found these lower levels to be persistent long term [36, 39]. Also as expected with weight loss, adiponectin levels rise, with studies showing sustained increases in levels up to 14 months postoperatively [9, 40]. 

### 3.2. Sleeve Gastrectomy

In the sleeve gastrectomy (SG) procedure, a partial gastrectomy is performed, in which the majority of the greater curvature of the stomach is removed and a tubular stomach is created (Figure 1(b)) [3]. The SG constitutes the upper abdominal component of the biliopancreatic diversion with duodenal switch (see below). SG induces weight loss by physical restriction of food intake, but a growing body of evidence supports several important hormonal mechanisms in SG-induced weight loss as well. The SG results in approximately 60–70% of excess body weight loss at 2 years, positioning it between the LAGB and RYGB in terms of weight loss efficacy [41]. 

 As most of the ghrelin producing cells of the stomach are removed with the partial gastrectomy, ghrelin levels are decreased from one day to at least 5 years postoperatively [34, 42, 43]. In addition to being a restrictive procedure, SG also results in more rapid delivery of nutrients to the small intestine; thus, GLP-1 levels have been shown to rise in response to a meal in SG patients [44, 45]. PYY appears to be increased postprandially, at a level comparable to RYGB [44–46]. 

Adiponectin increases and leptin decreases [47] following SG. A human study showed bile acids to be unaffected by SG, [48] but a rodent study suggested an increase [23]. GIP has not been evaluated after SG.

### 3.3. Roux-en-Y Gastric Bypass

The Roux-en-Y gastric bypass (RYGB) surgery is characterized by the construction of a small proximal gastric pouch separated from the stomach remnant, which remains *in situ* (Figure 1(c)) [3]. Drainage of food from this small stomach remnant is redirected via a gastrojejunal anastomosis, such that approximately 150 cm of proximal small intestine is bypassed. Excess body weight loss seen two years after RYGB is approximately 70% [49]. 

While the primary mechanism of RYGB is restrictive, there is a malabsorptive component as well. Furthermore, as delivery of nutrients to the intestine is not only hastened, but also delivered more distally into the jejunum at initial presentation of nutrients to the intestine, a number of hormonal changes are noted that are thought to play an important role in weight loss. Accordingly, there is a meal-induced increase in both GLP-1 and PYY following RYGB [46, 50, 51]. Postprandial bile acid levels increase after RYGB to the level of healthy lean controls, which may play a role in potentiating GLP-1 release [22]. Limited data suggest that OXM rises postprandially after RYGB [52, 53]. 

The effect of RYGB on ghrelin has been difficult to elucidate, with some studies showing decreased fasting levels, and others showing higher fasting levels, particularly later postoperatively. This inconsistency in findings may be ascribed to differences in assays [9], disruption of vagal innervation [54], dissimilarities in surgical manipulation of the fundus, and/or the effect of RYGB on ghrelin being transient [52]. Most studies examining meal-induced suppression of ghrelin have not found a significant change after RYGB [9]. Fasting [21, 24] and postprandial levels [20] of bile acids may be elevated after RYGB surgery.

GIP, secreted more proximally from the small intestine, has been shown in some studies to be decreased fasting and postprandially following RYGB, which may reflect the bypassing of the proximal intestine from which GIP is secreted [17, 37]. However, other studies have shown no effect of RYGB on GIP [55, 56]. Overall, the evidence suggests that GIP is unlikely to be a major player in RYGB associated weight loss [3, 10]. 

Leptin decreases and adiponectin increases after RYGB [9, 57]. The leptin decrease after RYGB is similar to that found after LAGB [36] as well as following equivalent weight loss with lifestyle modification [58]. Further, the leptin decrease is proportional to weight loss in both surgical procedures, suggesting that regulation of leptin secretion is not mechanistically related to a specific surgical method. 

### 3.4. Biliopancreatic Diversion

In the biliopancreatic diversion (BPD) procedure, a partial gastrectomy is performed, and a gastroileostomy is created with a long Roux limb and a short common channel [3]. The biliopancreatic diversion with duodenal switch (BPD/DS) is a variant of the BPD procedure whereby a gastric sleeve is created and the pylorus is intact (as opposed to creation of a gastric remnant in the BPD alone) (Figure 1(d)). The biliopancreatic diversion with or without duodenal switch (BPD/DS) is primarily a malabsorptive procedure, with a restrictive component as well. As the most drastic alteration in anatomy, the BPD+/−DS is also the most effective for weight loss, with two-year excess weight loss of approximately 78–82% [59, 60]. 

GLP-1 levels have been shown to be consistently elevated postprandially following BPD/DS, with some studies showing increased fasting levels as well, particularly emerging 3 months or more following the procedure [9]. The limited literature available suggests that PYY increases in both the fasting and postprandial states [61–63]. GIP is consistently decreased following BPD, reflecting the bypass of the proximal GIP-secreting small intestine [17].

The ghrelin response after BPD and BPD/DS has been varied amongst studies, with some showing decreased fasting levels, some no change, and some increased fasting levels [61, 62, 64–66]. Leptin levels are consistently decreased after BPD/DS [9], whereas adiponectin response has been variable [9, 67].

As no studies could be found describing either hormonal associations with weight loss success or failure after BPD, nor in regard to weight recidivism after BPD, this procedure will not be discussed further in the current review. 

## 4. Hormonal Changes Associated with Weight Loss Failure or Recidivism after Bariatric Surgery

The relative success of bariatric surgery, when measured in terms of weight loss, varies with procedure, with LABG resulting in the least weight lost and BPD/DS the most [4]. However, substantial heterogeneity in weight loss success is seen amongst patients having the same procedure. The factors responsible for variation in weight loss success are dependent on a multitude of factors, including psychological issues, dietary factors, psychosocial circumstances, and medical comorbidities [5, 6]. It is postulated that hormonal differences may play a role in the variations seen in weight loss success as well. 

There is currently no consistently accepted definition for weight loss failure after bariatric surgery. An excess weight loss of <50% has been considered to represent failure [68], whereas other studies have defined surgical failure as a failure to achieve a BMI of <40 kg/m^2^ or <35 kg/m^2^ [69]. Heterogeneity in the reporting of absolute percent weight loss versus percent of excess weight lost (EWL) between studies creates further difficulty in navigating this literature.

In general, the nadir weight loss seen following bariatric surgery is at approximately 12–16 months, with a small amount of weight regain following [1]. However, there is great variation in weight trajectory amongst individuals having the same procedure performed, with some patients able to sustain most of the weight loss, and others regaining much of the weight that was initially lost. Whether or not a patient experiences weight recidivism is also very likely multifactorial, with mental health issues, dietary issues, and hormonal factors postulated to be contributory [5, 6]. 

The very limited data currently available regarding the potential association of hormonal variations with weight loss failure or recidivism in the bariatric surgery population are summarized by procedure below. 

### 4.1. Laparoscopic Adjustable Gastric Banding

Few studies have examined whether there is a relationship with LAGB induced weight loss success and hormonal changes. The current balance of the literature does not point to any hormonal mediators or predictors of weight loss success or failure with LAGB. 

One study examined preoperative ghrelin levels and found that women with higher ghrelin levels prior to LAGB had similar weight loss results 2 years postoperatively, compared to women with lower preoperative ghrelin [70]; thus, preoperative ghrelin levels do not appear to be a predictor of successful weight loss induced by LAGB. Another study, which found an increased area under the curve (AUC) for ghrelin release in response to a meal challenge at 1 y after LAGB, found no correlation between ghrelin levels (fasting or AUC) and degree of weight loss [36]. Leptin decrease at 1 y after LAGB has been found to correlate with the degree of weight loss after LAGB. As this is seen with dietary induced weight loss as well, it is thought to be an effect of weight loss and not causative [36]. 

We are not aware of any literature regarding hormonal associations with weight recidivism following LAGB.

### 4.2. Sleeve Gastrectomy

As the SG is a newer procedure which has only recently been recognized as a standard option for bariatric surgery [71], there is little long term data available on the efficacy of SG and even less in regard to hormonal variations that may play a role in weight loss failure or recidivism. 

One study followed 26 SG patients through 5 years and found that %EWL was 55% at 5 years, with 19% of patients regaining >10 kg from their nadir weight loss [43]. Amongst those patients who regained weight, a slightly higher plasma ghrelin was seen; this did not reach statistical significance, which may have been due to the small sample size. A rodent study showed that serum bile acids correlated positively with weight loss success following SG [23].

### 4.3. Roux-en-Y Gastric Bypass

PYY appears to play a role in the degree of weight loss obtained following RYGB. One study found that patients in the highest quartile of %EWL at 6 weeks post-op had a higher postprandial PYY response compared to those in the lowest quartile [72] and that a higher PYY response at 6 and 52 weeks post-op predicted a larger %EWL at 33 months postoperatively. Further, the patients in the highest %EWL quartile had a slightly higher PYY response at 33 months compared to their own PYY response at 12 months, whereas the PYY response in the patients in the lowest quartile of %EWL had not changed from 12 months to 33 months post-op. This study also found that lower ghrelin levels at 6 weeks post-op were associated with a larger weight loss at 33 months, but this association became nonsignificant when PYY was taken into account. Thus, it appears that the prandial PYY response shortly after surgery may be a predictor of more successful long term weight loss following RYGB. 

The postoperative GLP-1 response to a meal appears also to play a role in successful weight loss following RYGB. A small study found that patients with the least amount of weight loss at approximately 2 years after RYGB had smaller prandial PYY and GLP-1 responses compared to the patients in the cohort with the best weight loss success [73].

A study which did not find any significant changes in levels of ghrelin in the fasting state or in response to a meal challenge at 1 year following RYGB also found no correlation between degree of weight loss and ghrelin levels [36]. However, in a rodent model, postoperative weight loss was correlated with the magnitude of decrease in ghrelin levels [74]. Similar to weight loss induced by lifestyle alterations, the degree of leptin decrease at 1 y after RYGB has been found to correlate with the degree of weight loss [36].

Interestingly, the cardiac hormone B-type natriuretic peptide appears to be increased following gastric bypass surgery and correlates directly with the degree of weight loss [75]. That BNP has been shown to induce lipolysis as well as slow gastric emptying, and absorption suggests that BNP may have an etiologic role in successful weight loss [76]. Amino-terminal pro-B-type natriuretic peptide (NT-proBNP), a byproduct of BNP production, has been shown to be positively correlated with adiponectin, though the nature of this relationship has not been established [76]. 

Approximately 20% of the patients regain substantial weight within 1–3 years following RYGB [77]; the mechanisms by which this occurs are poorly understood. It has been suggested that patients who experience weight regain may manifest a particularly powerful neuroendocrine-metabolic starvation response to their initial weight loss that favors metabolic energy conservation and weight regain [78]. In a rat model of RYGB, rats who regained weight were found not to have the severalfold increase in plasma PYY concentration that was evident in rats that had sustained weight loss success [79]. In another rodent study, plasma leptin levels decreased less in the rats who regained weight compared with those who had sustained weight loss; it was postulated that the ratio of PYY to leptin may be of greatest importance, with a lower ratio associated with failure to maintain weight loss [78]. 

## 5. Conclusion and Perspectives

Much work is needed in terms of identifying hormonal associations with weight loss failure and recidivism after bariatric surgery. In the future, with a better understanding of this complex arena, assessment of hormone status could potentially be helpful in understanding the hormonal contributors to a patient's postoperative weight loss failure or recidivism and potentially aid the clinician in utilizing appropriate targeted hormone therapy to help them achieve successful or sustained weight loss. However, given that it has also proven difficult to identify hormonal predictors of weight regain following dietary weight loss in free living humans [25], this task in the bariatric surgical population may prove difficult indeed. 

An area even less explored is whether preoperative hormonal predictors of postoperative weight loss success exist. Ideally, a hormonal panel could be collected preoperatively, and help to guide the surgical and clinical team in terms of counseling the patient regarding predicted weight loss success and even perhaps in terms of which surgical strategy would be ideal in the context of that preoperative hormonal milieu. 

Future research studies must consider not only the role of hormonal factors, but also how these hormonal factors may play into psychological, sociocultural, and genetic factors in weight loss failure or recidivism after bariatric surgery. Recent evidence suggests that RYGB strongly affects the hedonic regulation of food intake, potentially by altering taste preferences and the reinforcing value of food [80–82]; how this may tie in with hormonal changes is currently not well understood. Integrative interdisciplinary approaches exploring these complex interrelationships could potentially increase the explanatory power of such investigations.
